# Translation, and validation of Dysphagia Outcome and Severity Scale (DOSS): Swedish version

**DOI:** 10.1186/s13104-023-06637-z

**Published:** 2023-12-14

**Authors:** Klara Movander, Tove Larsson Palmquist, Patricia Hägglund, Liza Bergström

**Affiliations:** 1 Department of Health and Rehabilitation, Speech and Language Pathology Unit, Sahlgrenska Academy at the University of Gothenburg, Gothenburg, Sweden; 2 Department of Clinical Sciences, Speech-Language Pathology, Umeå University, Umeå, Sweden; 3 Remeo Stockholm, Torsten Levenstams Väg 8, Sköndal, Stockholm, Sweden; 4 Division of Neurology, Department of Clinical Sciences, Karolinska Institute, Danderyd Hospital, Stockholm, Sweden

**Keywords:** Assessment, Deglutition, DOSS, Oropharyngeal dysphagia, Rating, Swallowing

## Abstract

**Background:**

Swallowing dysfunction (dysphagia) significantly impacts patient and medical outcomes. In Sweden, there is no comprehensive outcome measure for dysphagia that incorporates holistic assessment and dysphagia impact on a person’s impairment, function and participation. The Dysphagia Outcome and Severity Scale (DOSS) was developed and validated (in English) and incorporates the World Health Organisation’s (WHO) aforementioned, International Classification of Functioning (ICF) aspects. This study translated then evaluated the validity and reliability of the Swedish version, DOSS-S.

**Method:**

Translation occurred based on WHO recommendations. The Content Validity Index (CVI) of the translated version (DOSS-S) was assessed twice by 11 (multi-professional) dysphagia experts. Criterion validity and rater reliability was calculated using 18 Speech Pathologists assessing patient cases from International Dysphagia Diet Standardization Initiative—Functional Diet Scale (IDDS-FDS) research.

**Results:**

Very high CVI values (0.96–0.99) for the *linguistic* correlation, and high CVI values (0.84–0.94) for *applicability* correlation were achieved. High criterion validity of DOSS-S with IDDSI-FDS was demonstrated (*r*_s_ = 0.89, p < 0.01), with very high inter and intra rater reliabilities (ICC > 0.90).

**Conclusion:**

The DOSS-S demonstrated very high validity values, and very high inter and intra rater reliability. This research contributes to improved dysphagia management by providing interprofessional dysphagia clinicians with a validated scale to identify patient progression, communicate dysphagia status between regions and countries, and document patient outcomes using an ICF framework.

**Supplementary Information:**

The online version contains supplementary material available at 10.1186/s13104-023-06637-z.

## Introduction

Oropharyngeal dysphagia is the term used to describe swallowing difficulties pertaining to the oral cavity and/or pharynx [1]. Within the adult population, oropharyngeal dysphagia is a symptom of many different medical diagnoses (e.g., stroke, traumatic brain injury, neurological diseases, dementia, and head and neck cancer) [2, 3] and is also associated with intensive care and tracheostomy [4]. Oropharyngeal dysphagia may result in complications such as aspiration pneumonia, malnutrition, dehydration [1]. To appropriately assess and treat dysphagia and to reduce dysphagia related consequences, valid and reliable assessments scales are essential.

Instrumental assessments, such as Flexible Endoscopic Evaluation of Swallowing (FEES) and Modified Barium Swallow (MBS), are considered the gold standard of dysphagia assessments. Several valid and reliable tools and rating scales are available to assist the clinician in determining dysphagia impairment and severity when using these instrumental assessments. This includes, but is not limited to, the Penetration-Aspiration Scale [5], and the Yale Pharyngeal Residue Severity Rating Scale [6], and Modified Barium Swallow-Impairment, MBS-Imp scale [7]. Such impairment-based scales assist the clinician in rating the swallow safety (penetration-aspiration), and also the efficiency of the swallow (pharyngeal residue), across different bolus consistencies and amounts. Valid and reliable scales are essential for standardising the assessment process and the rating of different dysphagia characteristics. This, in turn, allows for evidenced-based and impairment-specific interventions, such as (i) modifying foods/fluids or trialing swallow manoeuvres (compensatory techniques), and/or (ii) providing rehabilitation/training strategies to target the specific underlying pathophysiology (impairment) [8].

However, when assessing dysphagia, it is also important to consider, not only the impairment or disorder, however the greater range of dysphagia management influences. For optimal dysphagia management, the World Health Organisation’s (WHO) International Classification of Functioning, Disability, and Health (ICF) advocates for the medical diagnosis and impairment level to be supplemented with a measurement of functional activity and the patient’s ability to participate while considering environmental and personal factors [9].

In 1999, O ‘Neil and colleagues [10], noted that many scales assessing dysphagia predominantly investigated the *impairment* aspect to dysphagia and did not incorporate the many other influencing factors which impact a person’s functional activity or participation—which became more clearly articulated by the ICF framework in 2002 [9]. O’Neil and colleagues [10], identified the need to address the lack of functional and participation measures in dysphagia assessment and subsequently developed and validated the Dysphagia Outcome and Severity Scale (DOSS). The DOSS was established and evaluated using 135 MBS studies and 4 DOSS-trained SLPs. It is a 7-point rating scale, with a score of 1 representing a severe swallow dysfunction and oral intake level, while a score of 7 indicates a normal swallow function and oral intake. Each of the seven DOSS levels comprehensively describe: (a) the impairment, considering the oral and pharyngeal stage physiology such as safety (penetration/aspiration) and efficiency (pharyngeal retention); (b) functional aspects such as diet recommendations and need for swallowing strategies; (c) level of nutrition (alternative feeding aspects); and (d) level of supervision and independence with oral intake. Development of the DOSS required raters to not only consider (i) the MBS assessment results, however also (ii) the environmental and personal factors such as patient’s premorbid nutrition, cognition, acuity of dysphagia, current medical status and environment in terms of supervision/support available for the patient [10]. The DOSS is reported to therefore provide a holistic dysphagia outcome and severity scale for objective and patient-centered dysphagia management [10].

The DOSS is both valid and reliable [10] and furthermore has consistently been used internationally within research to validate/develop other dysphagia screening instruments, dysphagia questionnaires, and rating scales [11–15]. Results from these researchers indicate strong construct validity [11], criterion validity [12, 13], and fair [13] to very high reliability [10, 15].

Currently, in Sweden, there is no holistic dysphagia rating scale that considers structural/physiological impairment, along with functional activity and participation measurements, as recommended by O’Neill et al., [10] and the ICF model [9]. Therefore, this research investigated the translation and cultural adaptation of DOSS into Swedish (DOSS-S) and further studied the validity and reliability of DOSS-S.

## Method

### Study design

This multistep translation and validation study was conducted in Sweden. Ethical approval was granted by the Swedish Ethical Review Authority (Dnr 2020–05246).

### Participants and procedures

Translation was based on the WHOs guidelines for translating and adapting instruments with a focus on meaning and content rather than literal translation [16]. As per previous literature, the process was enhanced using a strong multi-step translation methodology. [17, 18]. Translation, validation, and rater reliability was conducted with different participants in three phases: (1) multistep translation and validation via an Expert Panel using the Content Validity Index (CVI) [19], (2) criterion validity analysis, and (3) rater reliability testing, see Additional file 2: Figure S1, Flow Diagram, with process further described below.

### Part 1: Multi-step translation and content validity

- Step 1. Forward translation (from English to Swedish) was conducted by authors KM and TLP with Swedish as first language, strong English language proficiency. Individual translation by each author was conducted first, with a subsequent comparison of the two translations, to then create a synthesised version (DOSS-S translation 1.0).
- Step 2. DOSS-S translation 1.0 was reviewed by an Expert Panel (n = 11) using CVI [19]. The Expert Panel consisted of nine speech and language pathologists (SLPs), one otolaryngologist and one radiologist, from six different regions within Sweden. Recruitment occurred via convenience sampling [20]. Inclusion criteria included (a) experience with dysphagia instrumental assessment, (b) ≥ five years clinical experience in dysphagia management, and (c) fluency in Swedish and with English proficiency. The Expert Panel participants had 6–31 years of experience in dysphagia management, 3–31 years of instrumental assessment experience.
  Validity, as per CVI [19], used the Expert Panel to review the Swedish DOSS-S version compared to the English version. Each translation of the seven DOSS-S levels were rated by the Expert Panel using CVI, considering (a) linguistic equivalence between the English and Swedish versions, and (b) the understandability and applicability of the Swedish translation considering the health care context and dysphagia management in Sweden [18, 19], see Table 1.

**Table 1:** Rating Scale for Content Validity Index (CVI)

CVI—Linguistic equivalence rating	CVI—Understandability/applicability rating
1 = no linguistic equivalence	1 = no understandability/applicability
2 = small linguistic equivalence	2 = little understandability/applicability
3 = fairly good linguistic equivalence	3 = fairly good understandability/applicability
4 = very good linguistic equivalence	4 = very good understandability/applicability
- Step 3. Review of DOSS-S 1.0. The authorship team reviewed the Expert Panel’s review. (Authorship team = two SLP Honours students, and two research supervisors with 9–23 years of dysphagia management experience and 6–22 years of experience in instrumental dysphagia evaluations, both VFS and FEES). Author PH has Swedish as a first language with strong English proficiency; LB has English as a first language with strong Swedish proficiency. Review and synthesis of the Expert Panel’s CVI ratings and feedback resulted in DOSS-S translation 2.0.
- Step 4. Back translation (Swedish translation 2.0 to English) was conducted by an independent translator (Registered Dietitian, 30 years’ experience, with English as a first language and strong Swedish proficiency). Back translation focused on content, meaning and cultural equivalence, not linguistic equivalence.
- Step 5. Review of Translation 2.0 occurred with all four authors considering the DOSS-S translation 2.0, the forward translation’s differences, weaknesses, and areas for improvement. The new version, DOSS-S 3.0, was subsequently formulated.
- Step 6. The second Expert Panel review used the same 11 experts to rate (CVI) and review the latest DOSS-S, as per review 1 and CVI protocol (Table 1).
- Step 7. Revision and final version of DOSS-S was established after the authorship team synthesised the comments, feedback and CVI from the Expert Panel’s second review. Lastly, each of the seven DOSS-S levels were reviewed to ensure consistency with descriptive information presentation for each dysphagia level, that is, (i) functional impact was described first, followed by (ii) oral dysfunction symptoms, then (iii) pharyngeal phase symptoms, See Additional file 1: Appendix 1, DOSS—Swedish (DOSS-S).

### Part 2: Criterion validity

Both criterion validity and rater reliability used published patient cases from the development of the International Dysphagia Diet Standardization Initiative (IDDSI) Functional Diet Scale [21]. Eighteen SLPs, from eight different regions, with 1–15 years of dysphagia experience, were recruited to assess the 10 patient cases using the finalised DOSS-S version. The 18 SLPs represented a range of different healthcare regions in Sweden (Skåne, Västra Götaland, Östergötland, Örebro, Stockholm, Västernorrland, Västerbotten) with one Swedish SLP recruited from Norway. The SLPs represented different clinical workplaces, including neurology, acute stroke, head and neck cancer, otolaryngology, rehabilitation centre, intensive care, pediatric dysphagia, and home rehabilitation. Fourteen SLPs had experience regarding instrumental assessment of swallowing, two had experience using DOSS. Recruitment occurred via convenience sampling [20], using the National Dysphagia network, other professional forums/social media. For criterion validity, results for the patient cases, as rated by the 18 SLPs using the DOSS-S, were compared with the published IDDSI Functional Diet Scale for the same 10 patients.

### Part 3: Rater reliability

Inter rater reliability was calculated using the above 18 SLPs rating the 10 patient cases with DOSS-S. For intra rater reliability, the same 10 patient cases (re-randomised) were sent to the 18 SLPs to re-rate using the DOSS-S two weeks after the initial DOSS-S rating. Data was missing for four SLPs, leaving a total of 14 SLP ratings available for intra rater reliability analysis.

### Statistical analyses

Statistical analysis was performed in the IBM SPSS Statistics version 26 with significance set at *p* < 0.05.

### Content validity

1. The Item-CVI uses the Expert Panels ratings of the DOSS-S seven items. Item-CVI is calculated using acceptable ratings (score of 3 or 4) whereby the sum of expert panelists who gave the same item an acceptable rating is divided by the total number of expert panelists (n = 11) [18, 19].
2. Scale-CVI /Average, describes the scale's content validity in its entirety, and is calculated by dividing the sum of all Item-CVIs by the number of items on the scale (n = 7) [18, 19, 22]. Statistical analyses interpretation [19, 22–24] is provided in Table 2.
3. The difference between the Expert Panels’ first and second ratings using the Scale-CVI/Average measure (using scores 1, 2, 3, or 4) was calculated using Wilcoxon signed-rank test.

**Table 2:** Statistical analyses interpretation

Statisical analysis used	Interpretation				
^a^Content validity using Item-Content Validity Index	≥ 0.79 Excellent content validity	0.70–0.79 Revision of item is recommended	< 0.70 Item should be excluded		
^a^Content validity using Scale Index/Average	≥ 0.90 Excellent content validity	< 0.90 Needs review			
^b^Agreement using Intraclass Correlation Coefficient	> 0.90 Excellent agreement	0.75–0.90 Good agreement	0.50–0.75 Moderate agreement	< 0.5 Poor agreement	
^c^Agreement using Kappa statistic	0.81–1.00 Very good agreement	0.61–0.80 Good agreement	0.41–0.60 Moderate agreement	0.21–0.40 Fair agreement	< 0.20 Poor agreement

Interpretations for (a) content validity using Item-Content Validity Index, (b) intraclass correlation coefficient, and (c) rater agreement

^a^Validity Index as per Polit et al., and Rodrigues et al., [19, 22];

^b^Agreement using Intraclass Correlation Coefficient as per Koo et al., [23];

^c^Agreement using Kappa statistic as per Altman, [24]

### Criterion validity

The correlation between the DOSS-S scores and the IDDSI-FDS patient scores used Spearman's correlation analysis. Table 2 provides statistical interpretation for Spearman’s correlation analysis.

### Inter and intra rater reliability

Intraclass Correlation Coefficient (ICC), with a confidence interval (CI) of 95%, was used using’two-way mixed’ model,’absolute agreement’, based on the mean value of the 18 assessments. For intra rater reliability, the weighted Kappa analysis was performed, CI = 95%. For inter rater reliability, the mean of all weighted Kappas across patient cases was calculated. To interpret ICC and Kappa values, see Table 2.

## Results

### Part 1: Translation and content validity

Results for (a) linguistic equivalence, and (b) understandability and applicability in a Swedish health care context, are reported in Table 3.

**Table 3:** Expert panel’s Content Validity Index (CVI) ratings

DOSS item	(1) CVI rating for linguistic equivalence			(2) CVI understandability/applicability rating		
	Review 1	Review 2	Difference rating 1–2 *p-value*	Review 1	Review 2	Difference rating 1–2 *p-value*
DOSS Level 7	0.91^*^	1.00^*^		0.91^*^	1.00^*^	
DOSS Level 6	0.82^*^	0.91^*^		0.64	0.82^*^	
DOSS Level 5	1.00^*^	1.00^*^		1.00^*^	1.00^*^	
DOSS Level 4	1.00^*^	1.00^*^		0.91^*^	0.91^*^	
DOSS Level 3	1.00^*^	1.00^*^		0.82^*^	1.00^*^	
DOSS Level 2	1.00^*^	1.00^*^		0.73	0.82^*^	
DOSS Level 1	1.00^*^	1.00^*^		0.91^*^	1.00^*^	
Scale-CVI (Average)	0.96	0.99	*p* = 0.622	0.84	0.94	***p* = 0.015**

The calculation of Item-CVI based on number of experts who rated each item as 3 or 4. *DOSS*  Dysphagia Outcome Severity Scale. * = Minimum recommended Item-CVI > 0.79, as per Polit et al., [19]. Minimum recommended Scale-CVI/Average = 0.90 or higher [19]

Bold value indicates the statistically significant

### Part 2: Criterion validity

The SLPs' dysphagia ratings using DOSS-S showed a high correlation with the previously published IDDSI-FDS results (*r*_s_ = 0.89, *p* < 0.01).

### Part 3: reliability

Rater reliability demonstrated very high agreement, see Table 4.

**Table 4:** Rater reliability

	ICC	Kappa
Intra-rater reliability (n = 14)	0.97	0.85
Inter-rater reliability (n = 18)	0.99 (CI 0.988–0.998)	0.77
*p-value*	*p* < 0.001	*p* < 0.001

*ICC*  Intraclass correlation coefficient, *CI*  Confidence interval. Weighted Kappa used to calculate intra rater reliability. Inter rater reliability calculated using the mean of all weighted Kappas across patient cases

## Discussion

This research presents data for the validity and reliability of the Dysphagia Outcome and Severity Scale – Swedish version. Outcomes from the current study, demonstrate a high-quality translation with excellent content validity (CVI > 90%), strong criterion validity (*r*_s_ = 0.89), and good–excellent agreement (inter rater reliability K = 0.77, ICC = 0.995; intra rater reliability K = 0.85, ICC = 0.97). These results, in terms of DOSS-S content validity, criterion validity and rater reliability are discussed further below.

### Content validity

Although results demonstrate excellent content validity for the DOSS -S, with both literal translation (linguistic correlation) and cultural adaptation (understandability and applicability), this is likely influenced by the multi-step translation process with several revisions ensuring improvements at each step. Similar results are reported by previous translation research demonstrating high validity using a multistep translation process [17, 18]. Of interest, is that, for the *linguistic correlation*, the improvement from Expert Rating 1 to 2, was not significant (*p* = 0.62), which is likely due to the already high CVIs prior to the second Expert Panel rating. For the cultural adaptation aspects (*understandability and applicability*), improvements from the first to second translation were, in fact, significant (*p* = 0.015) with all Item-CVIs reaching the acceptable cut-off value (> 0.79) by the final expert panel review.

### Criterion validity

The DOSS-S demonstrated strong correlation (*r*_s_ = 0.89) with the IDDSI-FDS scale, a functional diet scale also with established high validity and reliability [21]. Such results are congruent with previous research [11–13, 25] where the original DOSS (in English) has shown high – very high correlations/criterion validity when compared to other dysphagia measures.

### Reliability

In the current study, the DOSS-S when used by Swedish SLPs to rate previously published patient cases, demonstrated excellent inter and intra rater reliability. The high reliability results are similar to reliability testing from the original DOSS [10] and other research [15]. Furthermore, reliability results from the present study, are likely to be representative of the larger Swedish SLP population since the 18 SLPs recruited were heterogenic in years of experience (1–15 years) and from seven different healthcare regions in Sweden. The high inter rater reliability from the DOSS-S results in this study are congruent with reliability results from the original DOSS study [10], and research by Kidney et al. [15], however, diverge from reliability findings by Zarkada and Regan [14]. Upon further investigation, this difference is likely due to the variable methodology used within these aforementioned studies.

### Limitations and future directions

As with all research, the current study has its limitations. In terms of criterion validity, results were based on the ratings by 18 SLPs rating the 10 published cases from the validated IDDSI-FDS [21], methodology which may have influenced these results. It is possible, and expected, that a different correlation would be achieved if the DOSS-S was compared with another dysphagia rating or functional diet scale, a recommendation for future research. Rater reliability was also calculated using these 10 published IDDSI-FDS cases. Future research should investigate DOSS-S validity and reliability with (a) a greater number of patient cases, and (b) using instrumental assessments. Rasch analysis is also warranted. Additionally, DOSS-S training and calibration may be recommended prior to use.

## Conclusion

Results demonstrate a high-quality DOSS-S translation with high - very high content, criterion validity and rater-reliability. This research presents a valid, reliable and comprehensive dysphagia assessment scale which incorporates the WHOs ICF aspects such as impairment at the structural/physiological level, functional activity, and participation measures – not previously available for Swedish clinicians.

### Supplementary Information

**Additional file 1. Appendix 1, DOSS - Swedish (DOSS-S).** The translated (Swedish) version of the Dysphagia Outcome and Severity Scale (DOSS).

**Additional file 2: Figure S1. Flow Diagram.** Flow diagram depicting the process of DOSS translation from English to Swedish, and the validation and reliability testing of the DOSS-S.

## Data Availability

The datasets used and/or analysed during the current study are available from the corresponding author on reasonable request.
