# Supplementary material for: Translation, and validation of Dysphagia Outcome and Severity Scale (DOSS): Swedish version
Source: BMC Res Notes. 2023 Dec 14;16:369. doi: 10.1186/s13104-023-06637-z (PMC10720115; doi:10.1186/s13104-023-06637-z)
Supplement: Supplementary file 1 — Additional file 1. Appendix 1, DOSS - Swedish (DOSS-S). The translated (Swedish) version of the Dysphagia Outcome and Severity Scale (DOSS). [file 13104_2023_6637_MOESM1_ESM.pdf]

Dysphagia Outcome and Severity Scale - Swedish (DOSS-S)

Total peroral nutrition: Normalkost (utan anpassning)

Nivå 7: Normal sväljfunktion i alla situationer.
- Normalkost (utan anpassning)
- Behöver inga strategier eller extra tid

Nivå 6: Funktionell sväljning. Kompenserar självständigt.
- Normalkost (utan anpassning), funktionell sväljning
- Kan behöva extra tid under måltider
- Patienten kan ha lätt fördröjd oral eller faryngeal fas, retention eller spår av retention på den laryngeala delen av epiglottis men kompenserar eller sväljer rent, självständigt och spontant
- Ingen aspiration eller penetration vid någon konsistens

Total peroral nutrition: Anpassad kost med visst stöd

Nivå 5: Lätt dysfagi: Viss tillsyn. Kan behöva avstå från en konsistens.
Kan uppvisa ett eller flera av följande symtom:
- Lätt oral dysfagi med nedsatt tuggfunktion och/eller oral retention som rensas undan spontant
- Penetration ovanför stämbandsnivå vid en eller flera konsistenser eller till stämbandsnivå vid en konsistens, men som rensas undan spontant under sväljning*
- Aspiration vid enbart tunnflytande vätska, men med kraftig reflexmässig hosta som rensar helt och hållet
- Faryngeal retention som rensas undan spontant

Nivå 4: Lätt-måttlig dysfagi: Emellanåt behov av uppmaningar under måltid. Behöver avstå från en eller två konsistenser.
Kan uppvisa ett eller flera av följande symtom:
- Oral retention som rensas undan på uppmaning
- Aspiration vid en konsistens, med svag eller utebliven reflexmässig hosta
  - Eller penetration till stämbandsnivå med hosta vid två konsistenser
  - Eller penetration till stämbandsnivå med utebliven hosta vid en konsistens
- Faryngeal retention som rensas undan på uppmaning

Nivå 3: Måttlig dysfagi: Behov av fullständig stöttning, tillsyn eller tillämpning av strategier. Behöver avstå från två eller flera konsistenser.
Kan uppvisa ett eller flera av följande symtom:
- Måttlig oral retention som rensas undan på uppmaning
- Penetration till stämbandsnivå med utebliven hosta vid två eller flera konsistenser
  - Eller aspiration vid två konsistenser med svag eller utebliven reflexmässig hosta
  - Eller aspiration vid en konsistens med utebliven hosta samt penetration till stämbandsnivå vid en konsistens med utebliven hosta
- Måttlig faryngeal retention som rensas undan på uppmaning

Behov av alternativ nutrition

Nivå 2: Måttlig-grav dysfagi: Behov av fullständig stöttning eller tillämpning av strategier. Endast delvist oralt intag (sväljer minst en konsistens säkert med total tillämpning av strategier).
Kan uppvisa ett eller flera av följande symtom:
- Gravt oralt spill eller retention i den orala fasen, rensar ej undan eller behov av flera uppmaningar
- Aspiration vid två eller flera konsistenser, utebliven reflexmässig hosta, svag viljemässig hosta
  - Eller aspiration vid en eller flera konsistenser med utebliven hosta samt penetration till stämbandsnivå vid en eller flera konsistenser med utebliven hosta
- Grav faryngeal retention, rensar ej undan eller behov av flera uppmaningar

Nivå 1: Grav dysfagi: Noll per os: Sväljer inte någon konsistens på ett säkert sätt.
Kan uppvisa ett eller flera av följande symtom:
- Gravt oralt spill eller retention i den orala fasen, rensar ej undan
- Tyst aspiration vid två eller flera konsistenser, icke funktionell viljemässig hosta
- Grav faryngeal retention, rensar ej undan
- Ingen sväljning ses

*Kan ej observeras genom Funktionell endoskopisk Undersökning av Sväljningen (FUS), men genom videofluoroskopi (VFS/TVSS)

The Dysphagia Outcome Severity Scale - Swedish version (DOSS-S) is the translated version of: **The Dysphagia Outcome Severity Scale**. *Dysphagia*. 1999;14(3):139-45. O'Neil KH, Purdy M, Falk J, Gallo L. Translation by Klara Movander, Tove Larsson Palmquist, Patricia Hägglund, Liza Bergström via: Gothenburg University; Umeå University; Remeo, Stockholm; Karolinska Institute, Danderyds Hospital (KI DS).
