# Supplementary figures and images for: Translation, and validation of Dysphagia Outcome and Severity Scale (DOSS): Swedish version
Source: BMC Res Notes. 2023 Dec 14;16:369. doi: 10.1186/s13104-023-06637-z (PMC10720115; doi:10.1186/s13104-023-06637-z)

**Figure S1.** *Flow Diagram.*

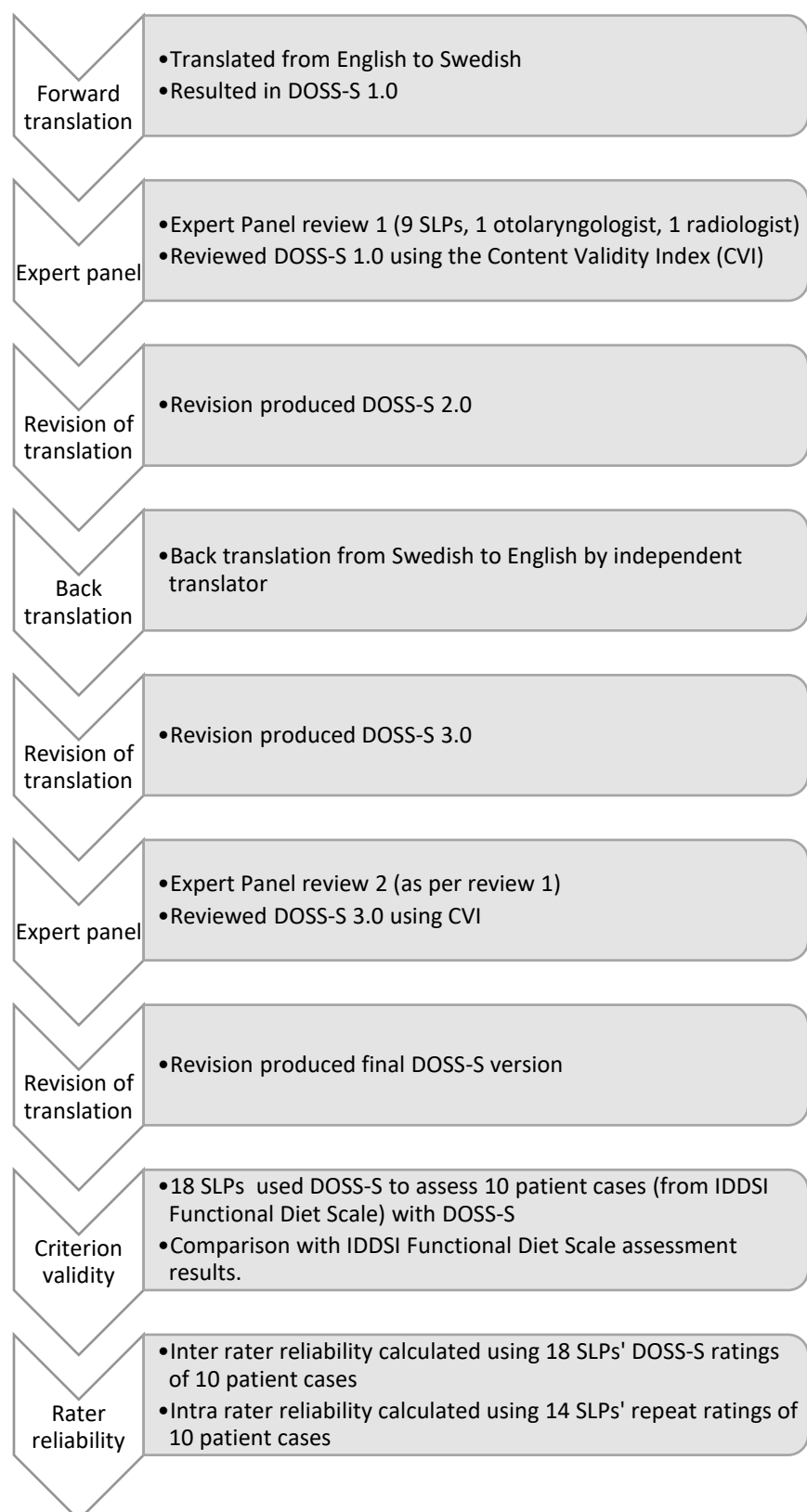

Supplement: Supplementary file 2 — Additional file 2: Figure S1. Flow Diagram. Flow diagram depicting the process of DOSS translation from English to Swedish, and the validation and reliability testing of the DOSS-S. [file 13104_2023_6637_MOESM2_ESM.pdf]
